# Simulation can offer a sustainable contribution to clinical education in osteopathy

**DOI:** 10.1186/s12998-019-0252-0

**Published:** 2019-07-10

**Authors:** Kylie M. Fitzgerald, Tracy Denning, Brett R. Vaughan, Michael J. Fleischmann, Brian C. Jolly

**Affiliations:** 1 College of Health & Biomedicine, Victoria University, Melbourne, Australia; 2 School of Health and Biomedical Sciences, RMIT University, Melbourne, Australia; 3 Department of Medical Education, University of Melbourne, Melbourne, Australia; 4 School of Medicine and Public Health, University of Newcastle, Callaghan, NSW Australia; 5 Adjunct Professor, School of Rural Medicine, University of New England, Armidale, NSW Australia

**Keywords:** Osteopathic medicine, Simulated learning, Directed observer

## Abstract

**Background:**

Clinical education forms a substantial component of health professional education. Increased cohorts in Australian osteopathic education have led to consideration of alternatives to traditional placements to ensure adequate clinical exposure and learning opportunities. Simulated learning offers a new avenue for sustainable clinical education. The aim of the study was to explore whether directed observation of simulated scenarios, as part replacement of clinical hours, could provide an equivalent learning experience as measured by performance in an objective structured clinical examination (OSCE).

**Methods:**

The year 3 osteopathy cohort were invited to participate in replacement of 50% of their clinical placement hours with online facilitated, video-based simulation exercises (intervention). Competency was assessed by an OSCE at the end of the teaching period. Inferential statistics were used to explore any differences between the control and intervention groups as a post-test control design.

**Results:**

The funding model allowed ten learners to participate in the intervention, with sixty-six in the control group. Only one OSCE item was significantly different between groups, that being technique selection (*p* = 0.038, d = 0.72) in favour of the intervention group, although this may be a type 1 error. Grade point average was moderately positively correlated with the manual therapy technique station total score (*r* = 0.35, *p* < 0.01) and a *trivial* relationship with the treatment reasoning station total score (*r* = 0.17, *p* = 0.132).

**Conclusions:**

The current study provides support for further investigation into part replacement of clinical placements with directed observation of simulated scenarios in osteopathy.

## Introduction

Clinical placements constitute a key element of all health professional programs, including osteopathy. The osteopathy course at Victoria University (VU) in Melbourne, Australia includes a mixture of classroom-based teaching and clinical placements. The osteopathy clinical education setting facilitates students application of skills, knowledge and attributes developed in the classroom to a real world setting [1]. There is currently a minimum number of hours and patient consultations that a student is required to undertake to complete each clinical placement unit [2]. However, increasing student numbers, and consequent clinical placement costs, necessitate development of alternative methods of delivering meaningful osteopathy clinical education. Simulation may facilitate clinical learning [3] and address some of these aforementioned issues [4]. Simulated learning environments (SLEs) are relatively new to osteopathy education but have been used in medicine for over 50 years [5–7]. Simulation is defined as “a technique, not a technology, to replace or amplify real experiences with guided experiences, often immersive in nature, that evoke or replicate substantial aspects of the real world in a fully interactive fashion” (pp. i2) [8]. Thus, simulation affords trainees the opportunity to safely practice clinical skills, procedures or routines in a SLE before actual patient exposure and may offer a strategy to bridge the gap between classroom and clinical education.

One of the benefits of simulation education is the ability to develop patient scenarios that align with learning outcomes and/or learning needs. For example, a SLE can be created to develop competencies in the management of uncommon scenarios or those where the risk to an actual patient may be too high. In terms of clinical placement activities, there is also an opportunity to combine both simulation and learning in the clinical environment whereby part-replacement of clinical hours or activities is through simulation.

One study reporting the results of two randomised controlled trials has supported the use of simulation to replace part of a traditional clinical placement for musculoskeletal clinical education in physiotherapy [9]. The control group completed a traditional four-week musculoskeletal clinical placement, while the experimental groups undertook training where 25% of their clinical placement was substituted with a SLE. The first experimental group undertook their four-week placement where the first week of their training was substituted with a SLE utilising simulated patients (SPs) (*n* = 192), and the remaining 3 weeks was via the traditional placement.

The second experimental group undertook a model that replaced 50% of the day with a SLE for the first 2 weeks of a four-week placement (*n* = 178) for students on a musculoskeletal rotation. The students undertaking either of the 25% simulated learning clinical replacement activities were assessed as being equally clinically competent as those undertaking the traditional clinical placements. However, the inclusion of live simulated patients in projects adds substantial human and financial costs that are limiting factors. A potential solution is for educational providers to include the concept of directed observing in simulation activities.

Directed observing is defined as learners being provided with active direction to observe a simulation without hands-on participation [4]. This systematic review of pedagogical methods, where the learner is positioned in an observer role, suggests use of observer tools such as performance checklist or feedback were associated with role satisfaction and achievement of learning outcomes. None of the studies cited in this review utilised video scenarios of simulated patients for learners undertaking their directed observer roles. The directed observer role could be adopted in video simulated learning activities as this offers a valuable learning experience without the additional costs or workload of live SP encounters.

Liu [10] compared 73 student physiotherapy and occupational therapy students undertaking a clinical scenario by either interacting with live simulated patient or watching a video of a simulated patient interacting with a clinician. There was no difference between the groups in their agreement with the expert clinician in identifying the patient problems. Participants in the video scenario group were more likely to agree with the expert clinician compared to the live simulated patient group. Student satisfaction with the simulated patient scenario was however significantly higher than those using the video scenario. The findings of this study provide some support for using video scenarios to assist students in determining a diagnosis and providing a treatment plan for a predetermined clinical case. The use of video recordings to support clinical learning warrants further investigation for achievement of clinical learning outcomes in osteopathy student learning [1].

Kneebone et al. [11] advocate for simulation to be used alongside clinical practice, and part replacement of clinical hours with simulation goes someway to addressing this goal. Given the identified issues with respect to clinical learning, the aim of the study was to evaluate whether directed observation of simulated scenarios could replace 50% of clinical placement time for year 3 osteopathy students and offer an equivalent learning experience prior as measured by their performance in an end of semester OSCE.

## Methods

This study was approved by the Victoria University Human Research Ethics Committee (HRE16–011).

### Participants

Participants were invited to volunteer to participate in the project via email in February 2016. Potential participants received a detailed information to participants leaflet that clearly outlined each step of the study, and that participants would be undertaking an experimental simulated learning alternative for part of their clinical placement. Potential participants were directed to contact the lead researcher for additional information or any questions.

#### Inclusion criteria

• Students enrolled in a year 3 full-time student load in the Bachelor of Science (Clinical Science) in 2016. Written consent was obtained from all participants.

#### Exclusion criteria

• Students undertaking a part-time load in the Bachelor of Science (Clinical Science) in 2016.

### Sample size

The sample size was limited by the number of students enrolled in year 3, semester 1, 2016 and eligible for the study (*n* = 76). There were also funding limitations for the facilitators providing marking and feedback for the experimental group (*n* = 10) which in turn limited the sample size for this group. The control group was 64 as 2 students did not sit the assessment determining the competence of students. To achieve a power of 80%, a sample size of 214 (intervention group = 29, control group = 185) would have been required and was not achievable with the resources available and student cohort size.

### Design

A non-equivalent group post-test only design was utilised [12]. Cook and Beckman [13] have advocated the use of post-test designs in medical education research, particularly where randomisation and appropriate sample sizes can be achieved. The nature of the current study design did not allow for true randomisation (participants self-selected into the intervention group) however the design overcomes threats to validity from a pre-test intervention [13]. Other authors have also used this study design with small participant numbers [14, 15]. Examiners involved in the OSCE were blinded as to the group allocation of the participants during the assessment however some were involved in teaching aspects of the program but not the simulation component.

#### Control group: CP (clinical placement) group (*n* = 64)

These participants completed their clinical placement hours in the VU Student Osteopathy clinic at the Flinders Lane campus. The control group participants attended a five-hour shift at the same time in a two-week block during semester 1, 2016 (Table 1). Participants completed six shifts of 5 hours, for a total of 30 clinical placement hours. Clinical placements allow the third year student to undertake a number of roles including reception duties and working with senior clinical students to assess treat and manage osteopathic patients [2]. Therefore the clinical placement enables the students to undertake both observation and hands on clinical skill application. Third year students interacted with the clinical supervisor in conjunction with the senior student leading the patient case, where each clinical educator is managing five senior students and up to five year 3 students. Participants had to complete 25 patient observations and complete a 500-word reflective report (graded pass or fail) as part of the academic requirements of the subject.

**Table 1:** Activity allocation for participants in semester 1, 2016

Group	Activity 1	Activity 2	Activity 3	Activity 4	Activity 5	Activity 6
Control (*n* = 64)	Clinic	Clinic	Clinic	Clinic	Clinic	Clinic
Experimental (*n* = 10)	Simulated Learning activity 1	Clinic	Simulated Learning activity 2	Clinic	Simulated Learning activity 3	Clinic

NB. Duration was five hours for each activity

#### *Experimental group: SL*+*CP Group – Simulated learning (50%)* + *Clinical Placement (50%) (n = 10)*

These participants were emailed a semester schedule for their clinical placement at the commencement of semester 1, 2016. The schedule (Table 1) stated when they had a clinical placement (50%, 3 × 5-h shifts = 15 h), and when they were required to undertake the directed observation of the simulated learning activities (50%, 3 × 5-h learning activities = 15 h). Thus, the experimental group had the opportunity to undertake the same number of learning hours as offered in the traditional clinical placement (control) group (30 h). This schedule was chosen as it mirrored the usual clinical placement schedule for year 3 students and enabled the participant’s time between simulated learning activities to review, reflect and plan their responses and receive feedback. The experimental group had to complete at least 12 patient observations (reduction of the control group requirements by 50% to account for the 50% reduction in clinical placement time). Participants did not have to complete the reflective report as each of the three cases had several opportunities for reflective writing.

### Intervention

All participants in the SL + CP group were added to a university online learning platform (Desire2Learn) with a module set up specifically for the study. This enabled students and facilitators online access to the video recordings of the simulated patient scenarios, associated questions and learning activities. Academic staff or students not participating in the intervention group did not have access to the module.

Students undertook directed observation of videos of simulated patient scenarios as it was more cost effective than using simulated patients in the osteopathy clinic. The cost of paying one actor to portray a simulated patient for each student per semester would be more than $30,000 and would be unsustainable. Creating videos of simulated patients interacting with expert clinicians in carefully planned and directed cases is a feasible and sustainable alternative [1]. This model may enable students to gain skills in clinical reasoning, diagnosis and treatment planning as directed observers without the ongoing expense of individual simulated patients. The videos can be used again which enhances the viability of this approach.

Each of the three online simulated learning activities included:

- A pre-task information page
- A set of learning outcomes
- Short videos of simulated patients acting out pre-written cases with an experienced osteopath showing each of the processes of:
  - ○ clinical history taking and communication
  - ○ examination
  - ○ clinical reasoning, and management
- Several short quizzes embedded at several points before or after question prompts
- A final ‘bring it all together’ discussion and short videos of the practitioner and patient reflecting on the history, examination and management.

The learning activities were mapped to the learning outcomes for the clinical and practical skill subjects that the students undertook in semester 1, 2016. With respect to the clinical scenarios, complexity was increased over the course of the semester to increase the level of challenge to students. The three activities were designed to commence with strong support from the facilitator and peers in the decision-making around the patient (first scenario) to some support (second scenario) to limited support so students were making decisions relating to the patient independently (third scenario). Participants then received detailed feedback from a trained facilitator as a group on the first two activities and individual feedback on the final activity.

### Measures

#### Comparison of pre-clinical academic performance of control group and experimental group

As the two groups of participants were being compared on academic criteria, each group’s grade point averages (GPA) prior to the commencement of the project (pre-clinical education) were obtained from the university student records system. Therefore, the means of the groups were compared to determine if they were academically equivalent before the study commenced.

#### Graded clinical competency measures

All participants undertook their scheduled end of semester clinical OSCE assessment in June 2016. The participants received a graded performance on a number of criteria within station two and three and were awarded a global rating for stations two and three (dependent variable).

The end of semester Objective Structured Clinical Exam (OSCE) had three stations:

1. Station 1 – Reading & Planning (ungraded): review structured case history and plan responses and techniques for stations 2 & 3
2. Station 2 – Oral Station: Osteopathic diagnosis & clinical reasoning (graded) where a student discusses likely diagnosis, alternative diagnoses and explains their model of clinical reasoning with possible treatment models to the examiner.
3. Station 3 – Oral and Practical station: High velocity low amplitude (HVLA) technique (graded) where the student identifies which techniques to use based on a set of written clinical findings, explains any safety issues and undertakes a structured informed consent process. Student then performs three HVLA techniques, one each to the spine, a junctional spinal region and a peripheral region on a model patient.

Participants received a score of 1–4 for the clinical competencies in station 2 & 3 (1 = Below expected level of performance, 2 = Borderline level of performance, 3 = Expected level of performance, 4 = Above expected level of performance). A score of 3 was the expected level of performance for each station component. All clinical competencies were mapped against the learning outcomes for the unit. Examiners were also asked to provide a global score of the competencies based on their overall impression of the student’s performance using the same score range.

### Statistical analysis

Data from each of the mark sheets were entered into SPSS (version 24) for analysis. Descriptive statistics were generated for each station item and the total score calculated. Correlation statistics were used to evaluate the relationship between the global rating and the total station score [16]. Inferential statistics were used to evaluate differences between any of the station item scores for group (ANOVA) and effect sizes calculated where relevant. GPA differences by group were evaluated using an independent measures t-test. A retrospective power calculation was also performed as the study was likely under-powered for these reasons (potentially identifying a type II error [17]) and to assist others with sample size estimates in future work [18].

## Results

The data met the assumptions for parametric inferential statistical analysis.

### Comparison of pre-clinical academic performance of groups

Academic performance prior to the commencement of the project was assessed using participant’s university calculated GPA. A significant difference with a large effect size (*p* = 0.048, d = 0.79) was observed with the simulation group (6.40 ± 0.36) having a higher GPA than the control group (6.04 ± 0.52).

### Comparison of performance on graded clinical competencies (end of semester clinical exams)

Correlations between the total score and global rating for both the Osteopathic Diagnosis & Clinical Reasoning station (*r* = 0.80) and HVLA Technique station (*r* = 0.79) were acceptable and suggested that the total station score accounted for approximately 62% of the global rating. Descriptive statistics for each item grouping and the station global and total scores are presented in Table 2. The only significant between-group difference was for Technique Selection in Station 3 (HVLA Technique) (*p* = 0.038, d = 0.72) with the experimental group having higher scores with a large effect size. A retrospective power calculation suggested the study was underpowered to detect a difference (51.7%) therefore the presence of a between-group difference may not reflect reality, and the effect size for this difference is likely to be inflated (type 1 error). No other criteria, nor the total score and global rating, were significantly different. GPA was positively correlated with the HVLA Technique station total score (*r* = 0.35, *p* < 0.01, *medium*) and reasoning station total score (*r* = 0.17, *p* = 0.132, *trivial*).

**Table 2:** Between group comparison of station performance

	Control Group	Experimental Group
	Mean +/− St Dev	Mean +/− St Dev
Station 2		
Diagnosis & Differential Diagnosis *(2 criteria)*	3.01 ± 0.44	3.05 ± 0.68
Clinical Reasoning (*3 criteria)*	3.17 ± 0.45	3.07 ± 0.41
Explanation of appropriate treatment models *(2 criteria)*	3.07 ± 0.42 s	3.13 ± 0.50
Global rating	3.21 ± 0.62	3.1 ± 0.56
Total score	21.51 ± 2.60	21.50 ± 2.83
Station 3		
Technique selection (1 criterion)*	3.09 ± 0.34	3.30 ± 0.48
Safety & Informed consent (3 criteria)	2.99 ± 0.36	3.03 ± 0.28
Performance of 3 HVLA techniques (3 criteria)	2.89 ± 0.55	2.76 ± 0.42
Global rating	2.92 ± 0.58	2.9 ± 0.32
Total score	20.65 ± 2.25	20.60 ± 0.84

*significantly different (*p* = 0.038, d = 0.72, *large*)

## Discussion

The current study sought to evaluate whether replacement of 50% of a learners’ clinical placement hours offered an equivalent learning experience as assessed by performance in the OSCE.

### Comparison of pre-clinical academic performance of groups prior to experiment

The pre-clinical academic performance as measured via GPA was found to be higher in the experimental group with a large effect size. This pre-clinical GPA included assessments in theory subjects such as anatomy, physiology, pathology and osteopathic theory. Students had also been assessed on a number of practical exams in anatomy and osteopathic examination and techniques in these pre-clinical years.

GPA is a measure of academic success by averaging grades across a course. The findings suggest the experimental group had a higher level of academic performance than the control cohort. In the context of the osteopathy course at VU the GPA only reflects student performance in theory subjects such as those assessed via test and written examinations. Examples include physiology, pathology, ethics and academic writing subjects. Practical osteopathy exams in the first two pre-clinical years are graded pass/fail and therefore do not contribute to GPA. The GPA does not represent the student’s academic performance in oral, practical and clinical skills. Therefore, without prior gradings in practical or oral exams for comparison, it is unclear whether the higher GPA achieved by the experimental group had any influence on this groups’ equivalent performance. This avenue could be explored in future studies where all practical assessments are graded in a curriculum.

Salem [19] demonstrated that preclinical GPA was both positively correlated with clinical GPA (*r* = 0.85) and a strong predictor of clinical GPA performance in 5th year medical students in Saudi Arabia. Results from the current study suggested that 12% of the variance in the HVLA Technique station score was associated with a learners’ GPA, however no association was observed for the reasoning station. The relationship between GPA and HVLA Technique station score may reflect assessment of knowledge within this station rather than psychomotor skill performance for these reasons. Salem [19] posits that learners’ with higher GPAs are likely to perform well in clinical performance assessments regardless of the clinical education model undertaken.

There is no apparent literature that has investigated whether students with higher GPAs are more likely to volunteer in educational research projects. Intrinsically motivated students have demonstrated higher GPAs [20] and it is possible the experimental group in the current study demonstrated higher intrinsic motivation however such an assertion requires evaluation. Future studies could explore the role of motivation in student research participation in clinical education and possible effects on the outcomes of health professions education research.

### Comparison of performance on graded clinical competencies (end of semester OSCE)

#### Station 2: osteopathic diagnosis and clinical reasoning station

There were no significant differences between the two groups overall performance on this station nor any differences at item level. This suggests the two groups were equivalent in the skills assessed in this station. From a clinical learning standpoint, this result reinforces the notion that participation in simulated learning did not have a detrimental effect on a learners’ clinical reasoning compared to their peers.

#### Station 3: HVLA technique

There were no significant differences between the participants group mean scores in the overall station score or for the items related to safety and informed consent, or practical HVLA skills. A significant difference with a large effect size was identified for the technique selection item in favour of the experimental group. The activity related to this assessment item required the student to provide a verbal rationale to the examiner for their choice of technique. The experimental group was better able to articulate their rationale or had a stronger understanding of the basis of the technique they were to apply.

The simulation scenarios included learning outcomes and question prompts designed to facilitate description of the participants reasoning processes. It may also be that these students were more self-confident as a link between competency and self-confidence is supported by the literature [21]. That said, consideration needs to be given to the underpowered and unbalanced nature of the study design and the possibility that the identified between-group difference is a statistical error rather than a real between-group difference in performance. Overall however, the results of both stations support replacing part of traditional clinical placement time with carefully structured simulated learning activities.

The final assessment items for this station (practical HVLA skills) are of particular importance as osteopathic education is traditionally based around a high number of practical skills classes and ‘hands-on practice hours’ supported by clinical placements. The simulated learning activities did not include any hands-on practice of osteopathy clinical skills and suggests that the replacement clinical placement hours were not detrimental to the participants continued acquisition and practice of their HVLA skills.

Simulation for manual therapy skill acquisition has been explored in osteopathy, predominantly for palpation skill development using haptic simulators [22, 23]. It is unlikely that an educator would hypothesize that a student would or should learn hands on skills, like HVLA technique, via video scenarios. The important factor here is that students were able to demonstrate equal competence in this clinical skill even though they spent less time in the traditional clinical placement. This suggests that undertaking the role of a directed observer instead of a portion of clinical placements may not be detrimental to their skill acquisition.

### Simulated learning activities versus traditional clinical placements

The finding that a percentage of traditional clinical placements can be partially replaced via simulated learning activities is supported by a variety of research [9, 21, 24]. Research in physiotherapy clinical education provides a comparator for the current work [9, 24]. Both studies found physiotherapy students undertaking the 25% simulation-based learning were as clinically competent at the end of the study as the students undertaking traditional clinical placements. Blackstock [24] used an RCT design with control and test groups of physiotherapy students in an acute care cardiorespiratory placement. The SLE involved 9 acute care scenarios with a different pathophysiology case for each. There were adequate sample sizes (*n* = 90 in each group) to achieve statistical power. The current study did not have the same sample size adequacy as there were external financial limitations on the number of participants that could be included in the experimental group.

There are some methodological differences between these studies and the current project. Watson [9] and Blackstock [24] both used live simulated patients the participants could interact with, not videos of the simulated patient encounters. Using live simulated patients (SP) was beyond the budget of the project as the cost of training and using an SP is approximately $300–400 per case. Therefore, the cost for 10 participants to undertake 3 clinical scenarios each would be $9000. This is clearly not a fiscally feasible or sustainable model without significant external funding. Using videos of the simulated patients was a method of sustainable development of educational resources. Future research could also explore the participant’s experiences with simulated learning qualitatively via focus groups.

There can be concerns from academic and clinical staff about replacing any clinical time with an alternative activity that does not involve direct contact with patients. Traditional clinical education models support experiential learning for practising and obtaining clinical skills through exposure to patients, however the nature of this environment may mean this is not always the outcome [25]. The results of this study as measured by the OSCE suggest both groups were equal in their clinical reasoning and manual therapy technique application. The simulated learning experience, on the basis of the results, appears to be equal to the traditional clinical placement and supports Kneebone et al. [11] in their advocacy of a mix of simulation and clinical practice.

However, this result should be considered with caution as there are a wide range of extraneous variables that have not been controlled or measured, such as influence of other learning activities or educators, differing numbers of patients while on clinical placement, extracurricular activities, part-time work and a ceiling effect with respect to the number of simulation sessions that may be needed to achieve equivalent competence. Future studies could consider the role these other factors might play. It is also important to note however there were significant costs involved in this study as live simulated patients, development of scenarios, supervision and feedback were significant initial expenses in this study. These expenses may not be justifiable or available in low resource learning environments however there may be opportunities to utilise lower cost options such as students from other courses (i.e. arts, media) who could use their participation as part of their own learning and assessment.

The sample sizes are unequal and future studies should consider having similar sized control and test groups although one must consider the ethical implications with randomly allocating students into one teaching approach versus another. These studies, if carried out at VU, will continue to be limited by the class sizes in osteopathy which are rarely greater than 80–90 students. Therefore, it could be worthwhile to continue this research with subsequent cohorts. Simulation research has also explored self-confidence and using the study design implemented here could be used to explore this concept and its translation into the clinical patient care environment. Further research could also explore how these students perform in their future clinical placements and workplace-based assessment results and their satisfaction with simulated learning approaches.

## Conclusion

Simulation affords an opportunity to part replace clinical placement hours where the simulation activities are aligned with those that would be undertaken during a clinical placement. The current study provides support for further investigation into part replacement of clinical placements with directed observation of simulated scenarios in osteopathy. Larger cohorts and randomisation of participants in future studies will strengthen these initial findings and provide evidence to support partial replacement.
